# Expression of Quaking RNA-Binding Protein in the Adult and Developing Mouse Retina

**DOI:** 10.1371/journal.pone.0156033

**Published:** 2016-05-19

**Authors:** Takahiko Suiko, Kensuke Kobayashi, Kentaro Aono, Togo Kawashima, Kiyoshi Inoue, Li Ku, Yue Feng, Chieko Koike

**Affiliations:** 1 Laboratory for Systems Neurosciences and Developmental Biology, Ritsumeikan University, Kusatsu, Shiga, Japan; 2 College of Pharmaceutical Sciences, Ritsumeikan University, Kusatsu, Shiga, Japan; 3 Graduate School of Life Sciences, Ritsumeikan University, Kusatsu, Shiga, Japan; 4 Center for Translational Social Neuroscience, Department of Psychiatry and Behavioral Sciences, Yerkes National Primate Research Center, Emory University, Atlanta, Georgia, 30329, United States of America; 5 Department of Pharmacology, Emory University School of Medicine, Atlanta, Georgia, 30322, United States of America; 6 Center for Systems Vision Science, Ritsumeikan University, Kusatsu, Shiga, Japan; 7 Precursory Research for Embryonic Science and Technology (PRESTO), JST, Chiyoda-ku, Tokyo, Japan; Universidade Federal do ABC, BRAZIL

## Abstract

Quaking (QKI), which belongs to the STAR family of KH domain-containing RNA-binding proteins, functions in pre-mRNA splicing, microRNA regulation, and formation of circular RNA. QKI plays critical roles in myelinogenesis in the central and peripheral nervous systems and has been implicated neuron-glia fate decision in the brain; however, neither the expression nor function of QKI in the neural retina is known. Here we report the expression of QKI RNA-binding protein in the developing and mature mouse retina. QKI was strongly expressed by Müller glial cells in both the developing and adult retina. Intriguingly, during development, QKI was expressed in early differentiating neurons, such as the horizontal and amacrine cells, and subsequently in later differentiating bipolar cells, but not in photoreceptors. Neuronal expression was uniformly weak in the adult. Among QKI isoforms (5, 6, and 7), QKI-5 was the predominantly expressed isoform in the adult retina. To study the function of QKI in the mouse retina, we examined *quaking*^*viable*^(*qk*^*v*^) mice, which have a dysmyelination phenotype that results from deficiency of QKI expression and reduced numbers of mature oligodendrocytes. In homozygous *qk*^*v*^ mutant mice (*qk*^*v*^/*qk*^*v*^), the optic nerve expression levels of QKI-6 and 7, but not QKI-5 were reduced. In the retina of the mutant homozygote, QKI-5 levels were unchanged, and QKI-6 and 7 levels, already low, were also unaffected. We conclude that QKI is expressed in developing and adult Müller glia. QKI is additionally expressed in progenitors and in differentiating neurons during retinal development, but expression weakened or diminished during maturation. Among QKI isoforms, we found that QKI-5 predominated in the adult mouse retina. Since Müller glial cells are thought to share properties with retinal progenitor cells, our data suggest that QKI may contribute to maintaining retinal progenitors prior to differentiation into neurons. On the other hand, the expression of QKI in different retinal neurons may suggest a role in neuronal cell type specific fate determination and maturation. The data raises the possibility that QKI may function in retinal cell fate determination and maturation in both glia and neurons.

## Introduction

*QkI* is a cloned gene lying immediately proximal to the deletion site in the *quaking*^*viable*^ (*qk*^*v*^) mutation on mouse chromosome 17. The dysmyelination phenotype of *qk*^*v*^ mutant mice is attributed to a deletion of a promoter element that drives *QkI* expression in myelinating glia[1–4]. The defects in maturation oligodendrocytes and myelin specific gene expression in the *qk*^*v*^ central nervous system (CNS) result in a reduction in the number of myelin lamellae produced and consequently the failure of the developing myelin to compact properly [5,6]. In addition, QKI has been shown to control glia cell migration and implicated in neural cell fate selection [7].

The *QkI* gene expresses 3 major alternatively spliced mRNAs (5, 6, and 7 kb) encoding QKI-5, QKI-6, and QKI-7, respectively, that differ in their C-terminal 30 amino acids [3]. All 3 QKI proteins are expressed strongly in myelin-forming cells and astrocytes, but absent in mature neurons in the adult brain, and individual isoforms show distinct intracellular distributions[8,9]. The QKI-5 isoform contains a nuclear localization signal, and can shuttle between the nuclear and cytoplasmic compartments[6]. QKI-6 can be detected in both the cytoplasm and the nuclei, and QKI-7 is predominantly cytoplasmic [10,11]. In *qk*^*v*^ mice, QKI-6 and QKI-7 are diminished in all myelin-forming cells, whereas QKI-5 is detected in the nuclei of Schwann cells as well as oligodendrocytes of less severely affected regions, such as hindbrain, cerebellum, and optic nerve [8]. Unlike *qk*^*v*^ mutant mice, ethylnitrosourea-induced mutants, *qk*^*k2*^ and *qkI*-deficient mice show early embryonic lethality as a result of abnormal vascular remodeling during embryogenesis [5,12]. These mutations demonstrate that the *qk* locus is pleiotropic, affecting diverse systems and implying that it defines some fundamental process employed by many tissues, and also suggest that QKI-5 is responsible for the lethality seen in *qkI-*deficient mice [13].

Based on the embryonic lethal phenotypes of mutant mice and recent findings of QKI function in various tissues, QKI is expected to be involved in many other fundamental processes besides myelinogenesis and oligogenesis in the CNS. QKI belongs to the evolutionally conserved STAR family of RNA-binding proteins, which are known as key regulators of biological processes that impact RNA metabolism [14]. QKI affects pre-mRNA splicing [10], mRNA turnover [15], translation [16], and miRNA regulation [17], and was recently shown to regulate one-third of the circular RNAs formed during the human epithelial-mesenchymal transition (EMT), suggesting that it plays specific biological roles in EMT [18].

The expression pattern and functions of QKI in the retina have not been investigated. To examine the spatial and temporal expression patterns of QKI during the development of the mouse retina, we employed immunohistochemistry. Retinogenesis in the mouse begins at embryonic day 11 (E11) and continues to postnatal day 14 (P14) [19]. During embryonic stages, retinal ganglion cells differentiate at E11, followed by the differentiation of horizontal cells, cone photoreceptors, and amacrine cells. During later retinal development, bipolar cells, rod photoreceptors, and Müller glial cells are born, mostly at postnatal stages; and retinal layers are specified at that time. We found that QKI was expressed predominantly in Müller glia in the adult retina. We further investigated QKI expression at P5 and P9, which are respectively the stages before and after retinal layer specification. QKI was expressed in Müller cells, and weakly in horizontal and amacrine cells, at P9 and thereafter. We also detected QKI protein in amacrine and horizontal cells of the P5 retina as well as in the differentiating bipolar cells. QKI was also found in retinal progenitors at embryonic stages. Based on our findings, we concluded that QKI may contribute to cell-fate determination or maturation of retinal cells, not only glial cells but also subsets of neuronal cells.

## Materials and Methods

### Animals

Retinas of embryonic and postnatal 129Sv/Ev mice for immunohistochemistry were used in this study. The mice were time mated, and embryos were designated as E0.5 at noon on the day the vaginal plugs were first observed. The expression studies were performed at least 3 times, each time with a different embryo or animal and at time points between E11.5 and P28 after euthanasia with carbon dioxide or cervical spine fracture dislocation. Retinas of embryonic and postnatal 129Sv/Ev mice for immunohistochemistry were used in this study. All animal procedures conformed to the Association for Research in Vision and Ophthalmology Statement for the Use of Animals in Ophthalmic and Vision Research, and were approved by the Animal Research Committee of Ritsumeikan University. The *qk*^*v*^ colony (Jackson Laboratory) was described previously [20]. Animal treatment for *qk*^*v*^ mice was according to National Institutes of Health regulations under the approval of the Emory University Institutional Animal Care and Use Committee.

### Immunohistochemistry

Retinas of postnatal mice were enucleated, and the vitreous was removed. The posterior retinal cups and embryonic heads were fixed in 4% paraformaldehyde in PBS (Nacalai Tesque, Kyoto, Japan) for 2 h for immunohistochemistry. Following fixation, samples were washed 3 times in PBS and then cryoprotected in 30% sucrose in PBS. To obtain sections, we embedded the samples in OCT medium (Tissue-Tek) and stored them at -80°C prior to sectioning at 20 μm for immunohistochemistry. The procedures for immunohistochemistry were described previously [21,22]. All analyses were performed with an LSM700 confocal microscope (Carl Zeiss, Oberkochen, Germany).

We used the following primary antibodies in this study: monoclonal antibodies specific for cyclin D3 (MBL, Nagoya, Japan), S-100ß (Sigma-Aldrich, St. Louis, MO), calbindin D28k (Swant Swiss antibodies, Switzerland), PKCalpha (Sigma-Aldrich, St. Louis, MO), Pax6 (Developmental Studies Hybridoma Bank, Iowa City, IA), HPC-1 (Sigma-Aldrich, St. Louis, MO), Brn3a (Merck Millipore, Billerica, MA), Ki-67 (BD Pharmingen, San Diego, CA), QKI-6 and QKI-7 (NeuroMab, Davis, CA) [23]. We used a rabbit polyclonal antibody against pan QKI (HPA019123, Atlas antibodies, Stockholm, Sweden) and QKI-5 (A300-183A, Bethyl laboratories, Montgomery, TX) [23]; and a sheep polyclonal antibody against Chx10 (Exalpha Biologicals Inc., Shirley, MA). Alexa secondary antibodies (Molecular Probes, Eugene, OR) were used at a concentration of 1:500; and TO-PRO-3 (Molecular Probes, Eugene, OR), at a concentration of 1:1000. Antigens detected in the various cell types by antibodies in the respective stages of development are described in the S1 Table.

### Immunodepletion

**Fig 1:**
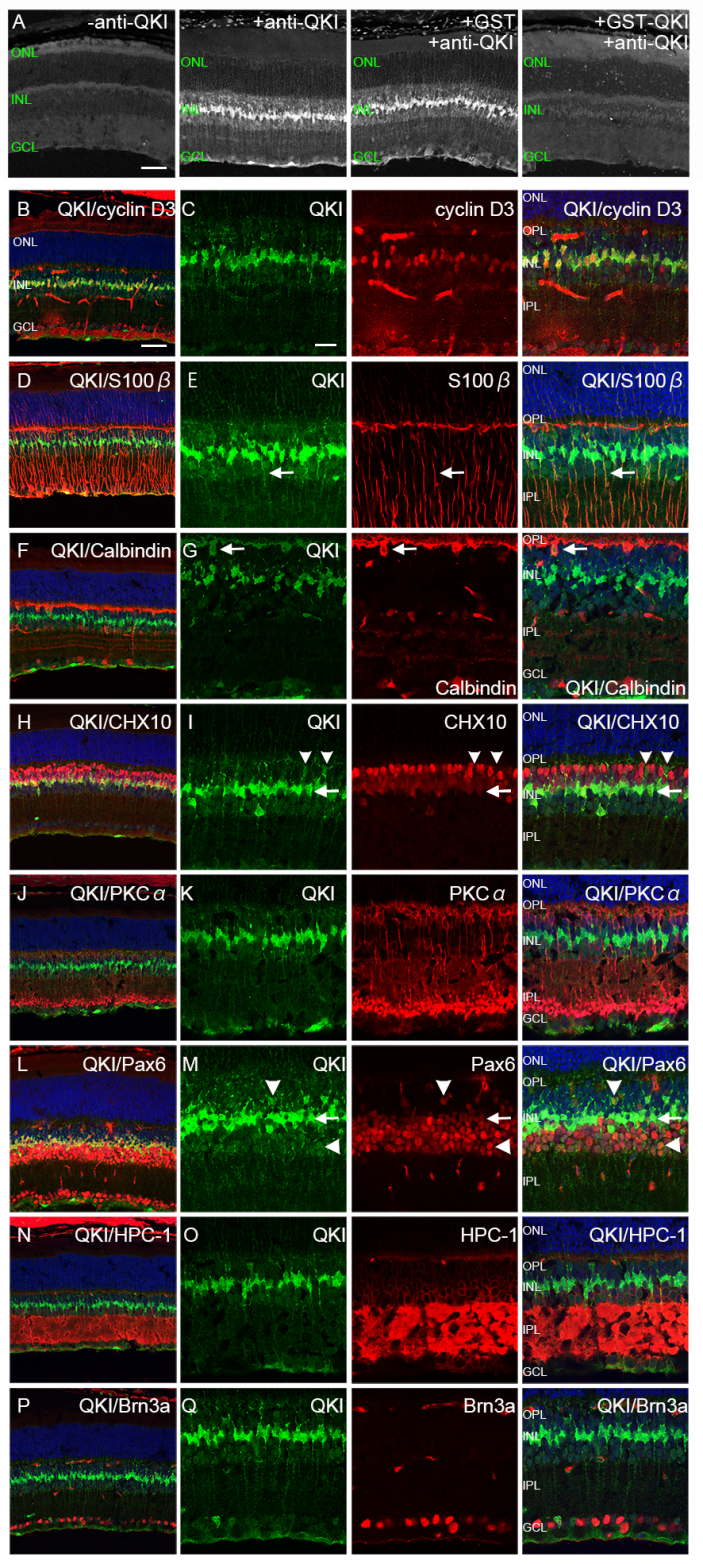
Characterization of QKI-expressing cells in the adult retina (P28). (A) Immunodepletion assay for QKI. Far left is the control without primary antibody. Immunostaining with anti-QKI antibody showed strong signals (middle left). Use of the anti-QKI antibody immunodepleted with GST–QKI (far right) resulted in significantly decreased signals, whereas the anti-QKI antibody incubated with GST showed strong signals by immunostaining (middle right). (B-Q) Retinal sections at P28 were double stained with anti-QKI antibody (green) and retinal markers (red). Nuclei were counterstained with TO-PRO-3 (blue). (B, C) QKI is expressed in the nucleus of Müller glial cells (cyclin D3). (D, E) QKI is expressed in the perinuclear soma of Müller glial cells (S-100ß, [arrows]). (F, G) Weak signals of QKI are expressed in horizontal cells in the INL (calbindin D28k, [arrows]). (H, I) Weak signals of QKI in the INL do not merge with strong Chx10 signals (arrowheads), whereas strong QKI signals merge with weak Chx10 signals (arrows). (J, K) QKI is not localized in the peripheral soma of ON bipolar cells (PKCalpha). (L, M) QKI signals co-localize with weak signals of Pax6 in the center of the INL (arrows), and weak signals of QKI in the inner and outer part of INL co-localize with Pax6 signals (arrowheads). (N, O) Weak signals of QKI in the inner layer of INL are surrounded by those of HPC-1, indicating peripheral soma of amacrine cells. (P, Q) QKI is not co-localized with ganglion cells (Brn3a). ONL, outer nuclear layer; INL, inner nuclear layer; GCL, ganglion cell layer; OPL, outer plexiform layer; IPL, inner plexiform layer. Scale bars are 50μm for A, B, D, F, H, J, L, N, and P and 20μm for C, E, G, I, K, M, O, and Q.

For the GST–QKI construct, we used a *QKI* 163–311 F primer (5'-GAATTCAGAGCAGAAATCAAGCTGAAG-3') and *QKI* 163–311 B primer (5'-TTTAAATAACACACCACTGGGTTC-3'). Cloned *QKI* was inserted into the EcoRI/SmaI sites of the pGEX6P'1 vector. For immunodepletion of the QKI antibody, GST–QKI (QKI amino acid residues 163–311, RAEIKLKRAVEEVKKLLVPAAEGEDSLKKMQLMELAILNGTYRDANIKSPALAFSLAATAQAAPRIITGPAPVLPPAALRTPTPAGPTIMPLIRQIQTAVMPNGTPHPTAAIVPPGPEAGLIYTPYEYPYTLAPATSILEYPIEPSGVL) protein and GST protein alone were isolated from 3 liters of an Escherichia coli (BL21 DL3) culture by use of a glutathione-Sepharose 4B column (GE Healthcare). The bound protein was eluted by adding 50 mM Tris-HCl and 10 mM reduced glutathione, pH8.0. QKI antibody was combined with approximately 5 times more GST-QKI or GST (molecular amount in PBS, and then the mixture was incubated at 4°C overnight and thereafter applied to the P28 retina (Fig 1, S1 Fig).

### Western blot analysis

Retinas of one month old *qk*^*v*^ mice were used for western blotting in this study. Dissected retina and optic nerve were lysed by sonication as previously described [20]. The protein concentrations of each sample were estimated from the intensity profile of total proteins on SDS-PAGE by coomassie staining. For detection, we used the polyclonal anti-QKI-5 antibody from Bethyl laboratories (Montgomery, TX) and monoclonal anti-QKI-6 and QKI-7 antibodies from NeuroMab (UC Davis, CA, USA)[23].

## Results and Discussion

### QKI was expressed predominantly in Müller glial cells and weakly in horizontal and amacrine cells in the adult mouse retina

Although QKI has been implicated in the axonal development of retinal ganglion cells [24], the expression of QKI in the retina had not been reported. We thus looked at the expression of QKI in the fully mature mouse retina (P28) by using immunohistochemistry to label retinal sections with anti-QKI antibody (Fig 1). QKI-expressing cells were observed in the mature retina (Fig 1A). To confirm the specificity of the anti-QKI antibody, we performed an immunodepletion assay (Fig 1A and S1 Fig). We produced and purified GST–QKI protein by employing the antigen sequence to raise an antibody, and used the GST protein alone for a control. Depletion of the anti-QKI antibody by use of GST-QKI for immunoabsorption eliminated the immunofluorescent signal when the mouse retina was immunostained for QKI, although the depletion using the GST control had no effect at all on the QKI immunostaining signal (Fig 1A and S1 Fig). Therefore, the anti-QKI antibody we used in the current study specifically detected QKI-expressing cells.

We detected a strong QKI signal at the central part of the inner nuclear layer (INL) and a weak signal at the inner and outer parts of the INL and the ganglion cell layer (GCL) in the P28 retina (Fig 1A). Next we characterized QKI expression by double labeling with anti-QKI antibody and antibodies against various retinal cell markers to identify the cell types expressing QKI (Fig 1B–1Q). First, we examined if the strong QKI signals at the central part of the INL came from Müller glia. Co-labeling with anti-cyclin D3 or anti-S100ß, which are markers for Müller glia [25–28], showed that QKI was predominantly localized in the nuclear or perinuclear soma, respectively, of Müller glia (Fig 1B–1E). We next characterized the weak QKI signals in the outer and inner parts of the INL with antibodies specific for retinal neuronal markers. Co-labeling with anti-calbindin, a horizontal cell marker [29], demonstrated that QKI was weakly expressed in horizontal cells (Fig 1F and 1G). We next co-labeled with Chx10, a pan bipolar cell marker [30] that is also expressed weakly in Müller glia [31], and found that weak QKI signals at the outer part of the INL did not colocalize with the strong Chx10 signals in bipolar cells; although Müller glial cells weakly expressing Chx10 gave strong QKI signals (Fig 1H and 1I). Co-labeling with PKCalpha, an ON bipolar cell marker [32], indicated that QKI was not expressed in PKCalpha-positive cells (Fig 1J and 1K). Taken together, these data suggest that QKI was not expressed in retinal bipolar cells. Co-labeling with antibody against Pax6, a marker for progenitor cells and also for ganglion cells, amacrine cells, horizontal cells, and Müller glia in the mature retina [33], showed that weak QKI signals in the outer and inner parts of the INL were detectable in Pax6-positive cells that were supposedly horizontal and amacrine cells, respectively (Fig 1L and 1M [arrowheads]). Strong QKI signals in faintly Pax6-positive cells in the central part of the INL were presumably from Müller glial cells (Fig 1L and 1M [arrows]). Co-labeling with anti-HPC-1 (Syntaxin), an amacrine cytoplasmic marker [34], showed that faint QKI-positive cells were surrounded by perinuclear HPC-1 signals in the INL (Fig 1N and 1O), again indicating weak QKI expression in amacrine cells. Co-labeling with Brn3a, a ganglion cell marker [35,36], showed that QKI was not expressed in retinal ganglion cells (Fig 1P and 1Q). Taken together, these results demonstrate that QKI was expressed predominantly in Müller glial cells, and weakly in horizontal and amacrine cells in the adult retina.

### QKI was expressed in differentiating retinal neurons in the postnatal developmental stages

**Fig 2:**
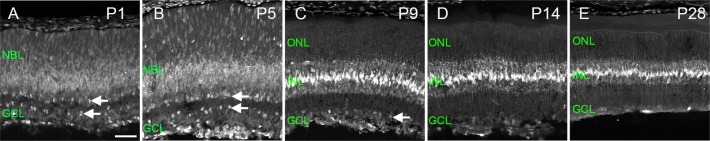
Localization of QKI in the postnatal developing retina. (A-E) Retinal sections at P1 (A), P5 (B), P9 (C), P14 (D), and P28 (E) were stained with anti-QKI antibody. Arrows show dotty signals in the inner NBL (A, B) and GCL (A, B, C). Scale bars are 50 μm.

We next examined the localization pattern of QKI in the postnatal developing retina by using anti-QKI antibody (Fig 2). QKI-positive cells were distributed in the NBL of P1 and P5 retinas, in addition to their dispersed presence in the inner part of the NBL and near the GCL (Fig 2A and 2B [arrows]). QKI expression became restricted to the INL and GCL after P9 (Fig 2C–2E). Since the expression pattern differed before and after retinal layer specification, we set out to determine which types of neuronal cell express QKI, besides the Müller glia, in the differentiating retina at P5 and P9.

**Fig 3:**
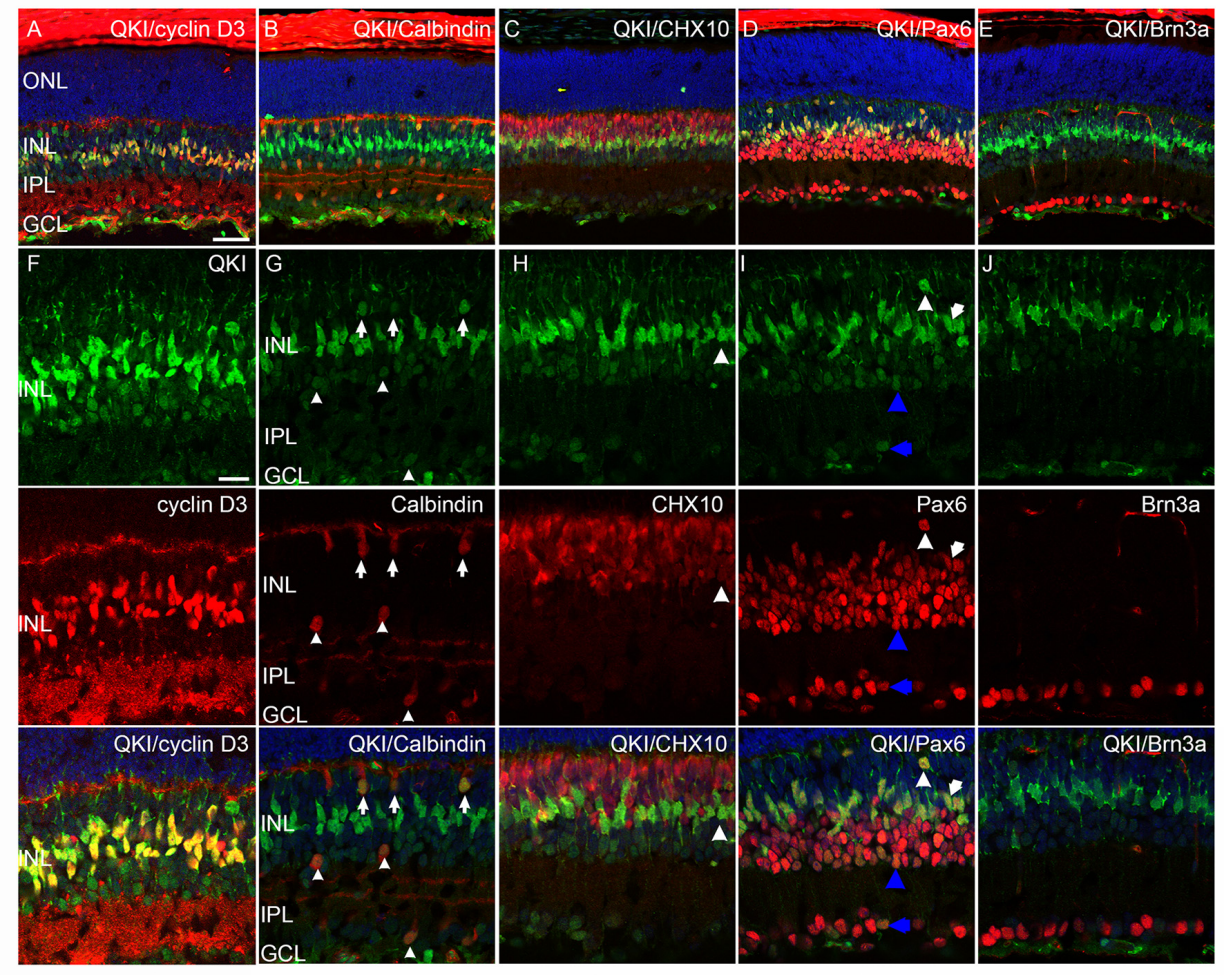
Characterization of QKI-expressing cells in the P9 retina. (A-J) Retinal sections at P9 were double stained with anti-QKI antibody (green) and antibodies against other retinal markers (red). Nuclei were counterstained with TO-PRO-3 (blue). (A, F) Strong QKI signals merge with strong cyclin D3 signals. (B, G) Weak QKI signals merge with calbindin at the outer INL (arrows) and outer and inner IPL (arrowheads). (C, H) Strong QKI signals merge with weak Chx10 signals (arrowheads). (D, I) All QKI signals merge with Pax6 in the P9 retina. Double-positive cells indicated with white arrowheads, white arrows, blue arrowheads, and blue arrows are likely horizontal, Müller glial, amacrine cells, and a subset of ganglion cells, respectively. (E, J) Weak QKI signals in the GCL do not merge with Brn3a. Scale bars are 50 μm for A-E and 20 μm for F-J.

**Fig 4:**
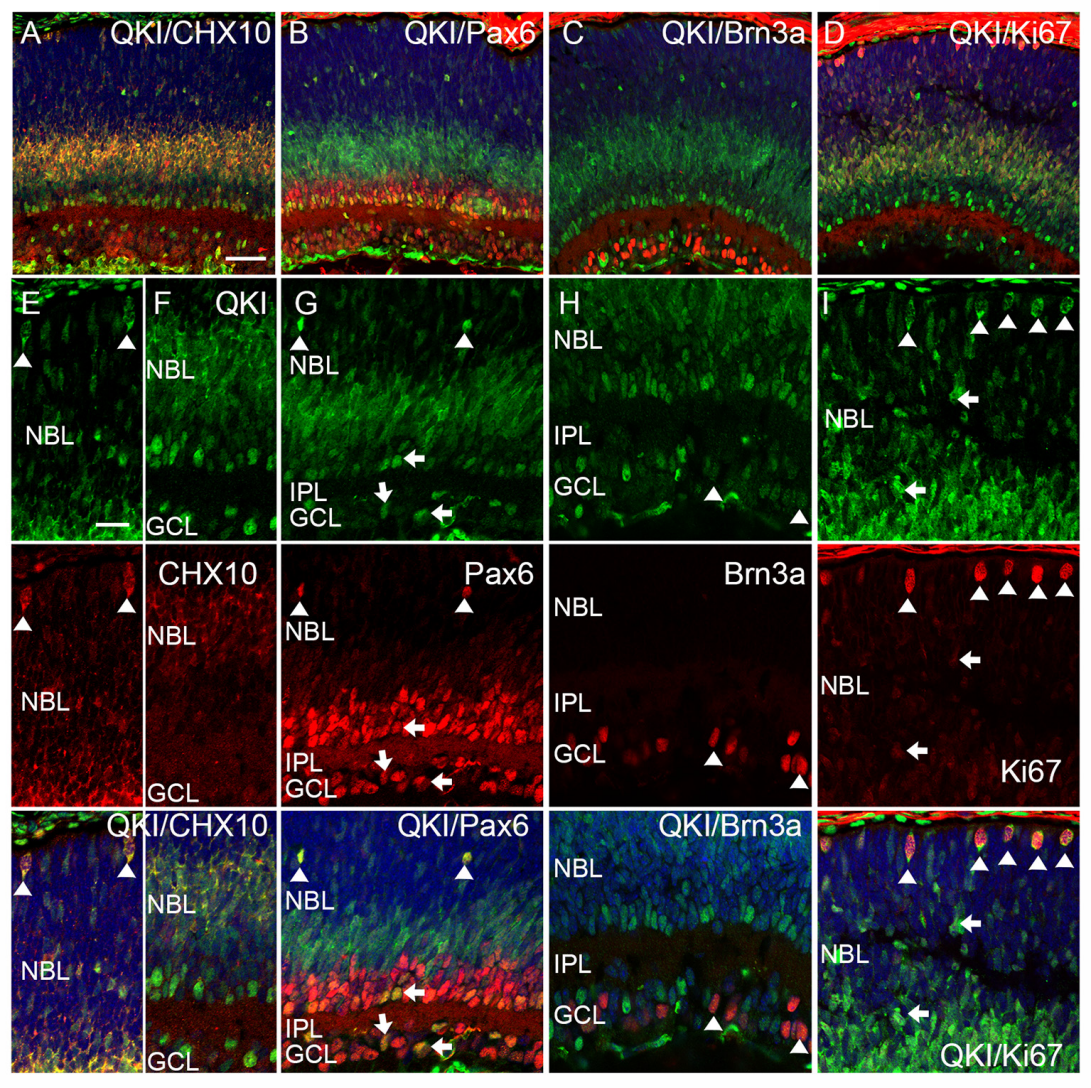
Characterization of QKI-expressing cells in the P5 retina. (A-I) Retinal sections at P5 were double stained with anti-QKI antibody (green) and antibodies specific for other retinal markers (red). Nuclei were counterstained with TO-PRO-3 (blue). (A, E, F) QKI signals at the apical edge (E, arrowheads) and inner part (F) of the NBL merge with Chx10. (B, G) QKI-positive cells in the outer NBL and in the peripheral IPL express Pax6 signals (arrowheads and arrows, respectively). (C, H) Some weak QKI signals in the GCL merge with Brn3a signals (arrowheads). (D, I) QKI signals are detected in strongly and weakly Ki67-positive cells (D, I, arrowheads and arrows). Scale bars are 50 μm for A-D and 20 μm for E-I.

First we investigated the P9 retina with anti-QKI antibody and antibodies against retinal nuclear markers (Fig 3). The Müller glial marker Cyclin D3 is expressed during the late postnatal stages but not in the early stages [25]. Strong QKI signals in the P9 retinas colocalized with cyclin D3 signals in the central part of the INL (Fig 3A and 3F). Interestingly, faint QKI signals were detected with calbindin signals at the outer part of the INL (arrows) and outer and inner parts of the inner plexiform layer (IPL) (arrowheads), indicating that QKI was expressed in the horizontal cells and in a subset of amacrine cells, respectively (Fig 3B and 3G) [29]. Weak Chx10-expressing cells showed strong QKI signals in the center part of the INL, which were also detected in cyclin D3-positive cells (Fig 3A), indicating Müller glial cells (Fig 3C and 3H [arrowheads]) [37]. Interestingly, almost all of the QKI signals in the P9 retina were detected in Pax6-positive cells in the INL, indicating the expression of QKI in horizontal, amacrine, and Müller glia (Fig 3D and 3I). We also detected QKI and Pax6 double-positive cells in the GCL (Fig 3I); but QKI signals were not co-localized with Brn3a (Figs 4E and 3J), suggesting that QKI-positive cells in the GCL could have been displaced amacrine cells. Taken together, our data indicate that the cells expressing QKI in the P9 retina were not different from those in the P28 retina (Fig 1). Therefore, these data demonstrate that QKI was expressed in Müller glial cells and early differentiating neurons, such as horizontal and amacrine cells, at P9 and that its expression was maintained during and after maturation.

Next we investigated the P5 retina by co-labeling retinal sections with anti-QKI antibody along with antibodies against retinal neuronal nuclear markers and a proliferating cell marker, Ki67 (Fig 4) [38]. Cyclin D3 and calbindin were not detected at P5 (data not shown). Chx10 is expressed in both bipolar cells and progenitor cells [37]. Most of the QKI signals in the NBL colocalized with Chx10 signals, suggesting that QKI was expressed in developing bipolar cells (Fig 4A and 4F). A few of the Ki67 and QKI double-positive cells in the NBL were likely progenitor cells (Fig 4D and 4I [arrows]). QKI signals at the apical edge of the retina colocalized with Chx10 and Ki67 signals, indicating that these cells were progenitors at mitotic stages (Fig 4A, 4D, 4E and 4I [arrowheads]). Intriguingly, QKI-expressing cells did not completely match Pax6-positive cells in the P5 retina (Fig 4B and 4G). Most of the QKI signals in the NBL did not colocalize with Pax6 signals, but with differentiating Chx10-expressing bipolar cells (Fig 4A, 4B, 4F and 4G). Dispersed QKI-positive cells expressing Pax6 signals at the outer part of the NBL are consistent with horizontal cells (Fig 4G [arrowheads]). In the IPL and GCL, some of the QKI-positive cells expressed Pax6 (Fig 4B and 4G [arrows]). However, QKI-positive cells in the GCL were not significantly positive for the ganglion cell marker Brn3a; and, therefore, QKI and Pax6-positive cells were likely a subset of amacrine cells (Fig 4B, 4C and 4G [arrows], H [arrowheads]). Taken together, these results demonstrate that QKI was expressed in the proliferating cells and in differentiating bipolar cells at early postnatal stages.

### QKI was expressed in the proliferating cells throughout the embryonic stages of the mouse retina

**Fig 5:**
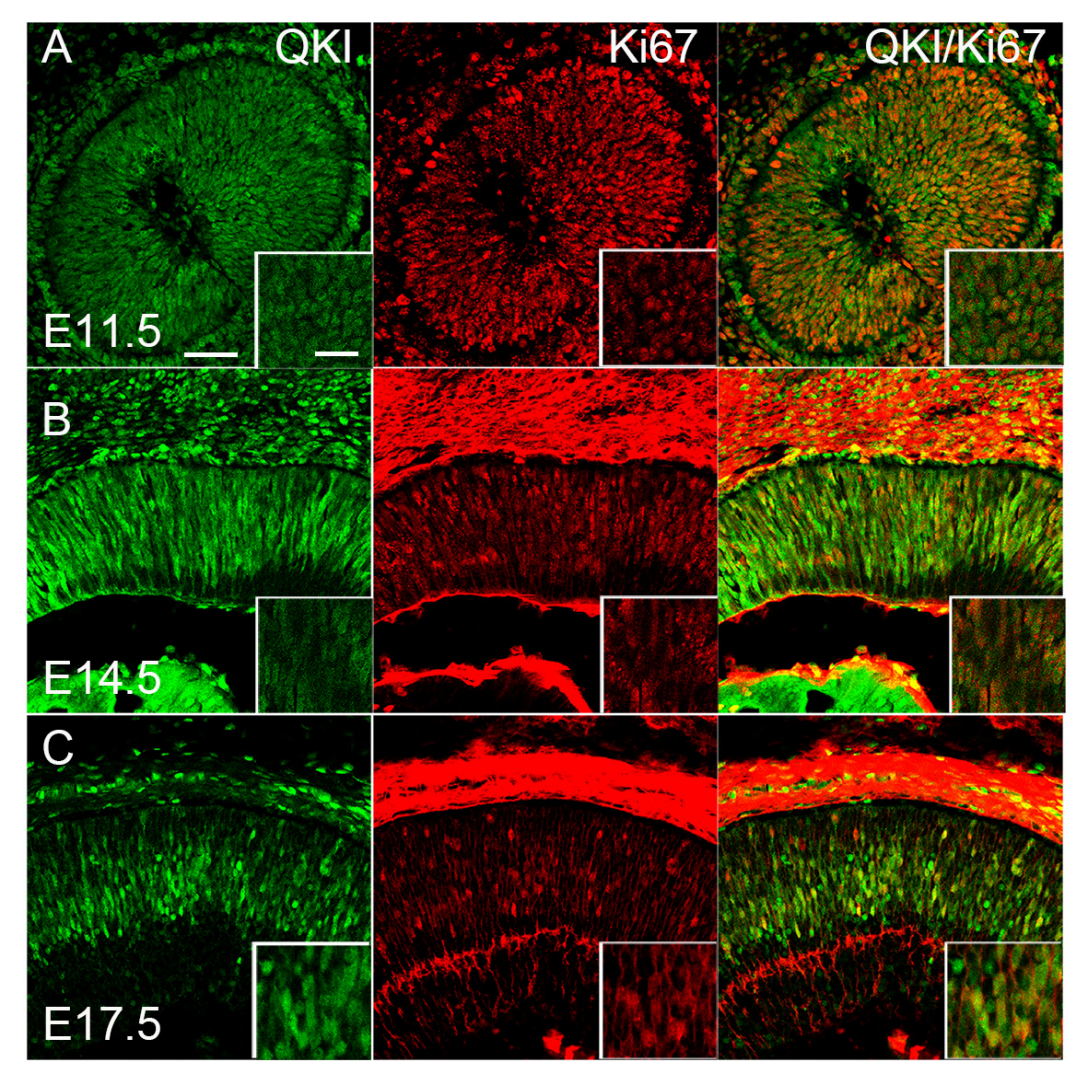
Characterization of QKI-expressing cells in the embryonic retina at E11.5 (A), E14.5 (B), and E17.5 (C). Retinal sections were double stained with anti-QKI (green) and anti-Ki67 (red) antibodies. Scale bar is 50 μm, and 20 μm for inset.

Since QKI was expressed in proliferating cells at early postnatal stages, we next examined its expression during embryonic stages. We examined the localization of QKI signals in the developing retina during embryonic stages by co-labeling retinal sections with antibodies against QKI and Ki67 (Fig 5). QKI signals were detected in the nucleus of Ki67-positive cells, suggesting that QKI was expressed in the retinal progenitors; although its distribution did not completely overlap with that of Ki67 signals in the same nucleus (Fig 5A–5C).

### QKI-5 is the predominantly expressed QKI isoform in the adult retina

**Fig 6:**
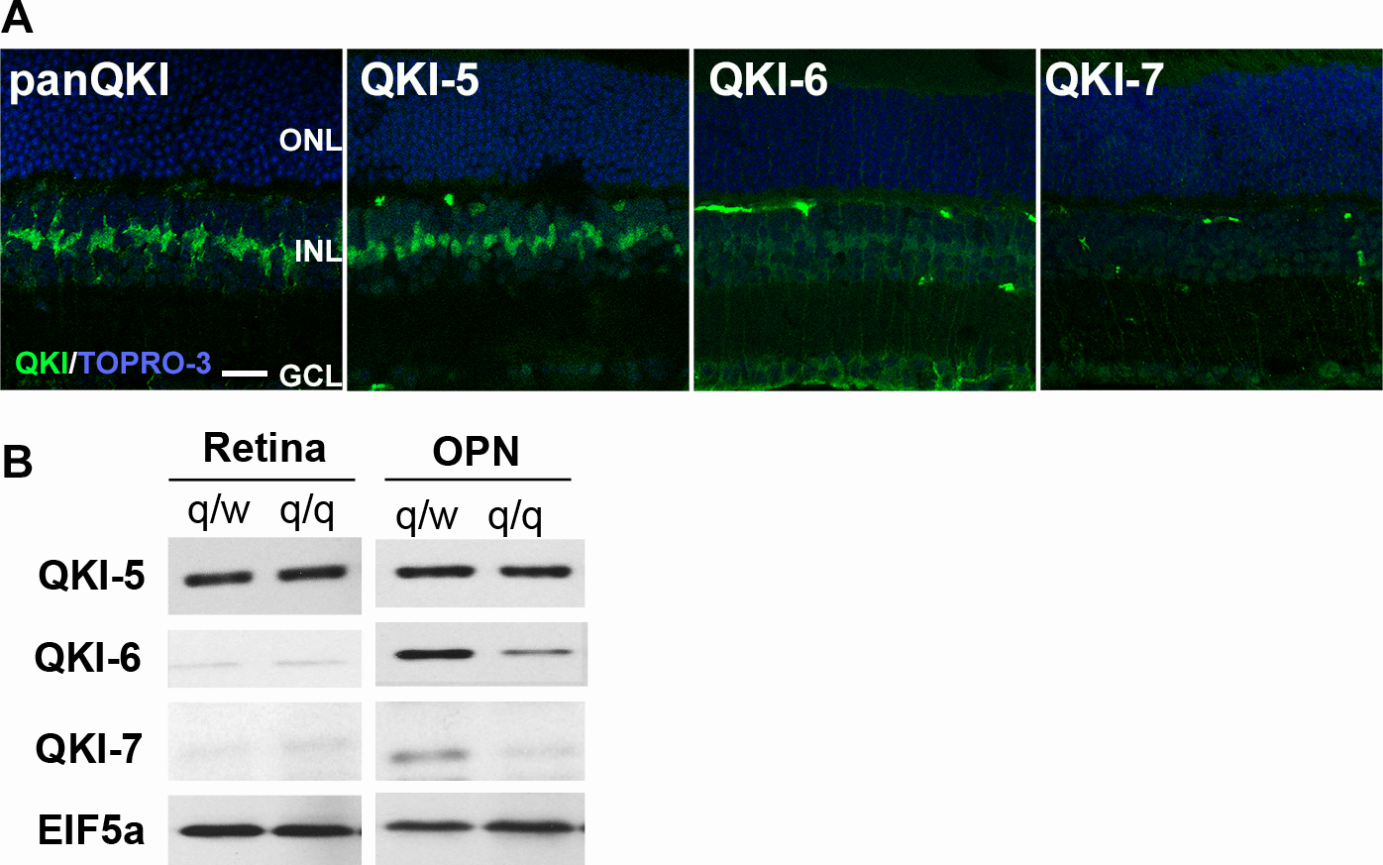
Immunohistochemistry of QKI isoforms in the wild type retina (A). Retinal sections at P28 were examined with pan anti-QKI, anti-QKI-5, -6 and -7 antibodies (green), respectively. Nuclei were counterstained with TO-PRO-3 (blue). Scale bar is 20 μm. Western blots of QKI isoforms in the retina and optic nerve in q/q mutant mice and q/w controls (B). There was no significant reduction of QKI-5 in both the retina and optic nerve (OPN). QKI-6 and QKI-7 were reduced in the OPN of q/q mice, while already low levels in the retina were not discernably reduced.

Mouse QKI has 3 isoforms resulting from splice variants (QKI-5, -6, -7) [5]. We studied the expression and localization patterns of all QKI isoforms in the adult mouse retina (P28) by immunohistochemistry (Fig 6A). Using isoform-specific antibodies [23], we obtained a prominent signal for QKI-5 in the retina. The localization of QKI-5 signal was similar to that detected by the pan anti-QKI antibody which was also used in Fig 1 and Fig 2E. An antibody against QKI-6 showed only dim labeling consistent with weak expression in Müller cell nuclei and cytoplasm. An antibody specific for QKI-7 did not detect any appreciable signal in the retina.

Because QKI-deficient mice are embryonic lethal at an early stage, we attempted to use the *qk*^*v*^ mutant to study the contribution of QKI in the retinal development. We first analyzed the expression levels of QKI isoforms in the *qk*^*v*^ mutant retina by western blot using the optic nerve as a control (Fig 6B). In the optic nerve of *qk*^*v*^*/qk*^*v*^ mutant mice (q/q), the expression levels of QKI-6 and -7 were markedly reduced as compared to the *qk*^*v*^/*wt* control (q/w) due to defects of QKI in myelinating oligodendrocytes whereas the levels of QKI-5 were unaffected. In contrast, in the q/q retina, we did not detect reduction of the predominant QKI5 isoform or the weakly expressed QKI-6 and QKI-7 isoforms. The continued expression of QKI in q/q mouse retina did not recommend the *qk*^*v*^ mutant for further studies on retinal anatomy and function. It is known that the reductions of QKI isoforms in q/q mouse are detected in oligodendrocytes but not astrocytes[8]. Thus, unlike *qk*^*v*^ oligodendrocytes in which QKI-6 and QKI-7 are preferentially diminished, the genetic lesion in *qk*^*v*^ mice does not affect QKI expression in the retina, which supports the hypothesis that the *qk*^*v*^ deletion possibly affected a promoter and/or enhancer that drives expression of the *qkI* gene specifically in oligodendrocytes [39].

**Fig 7:**
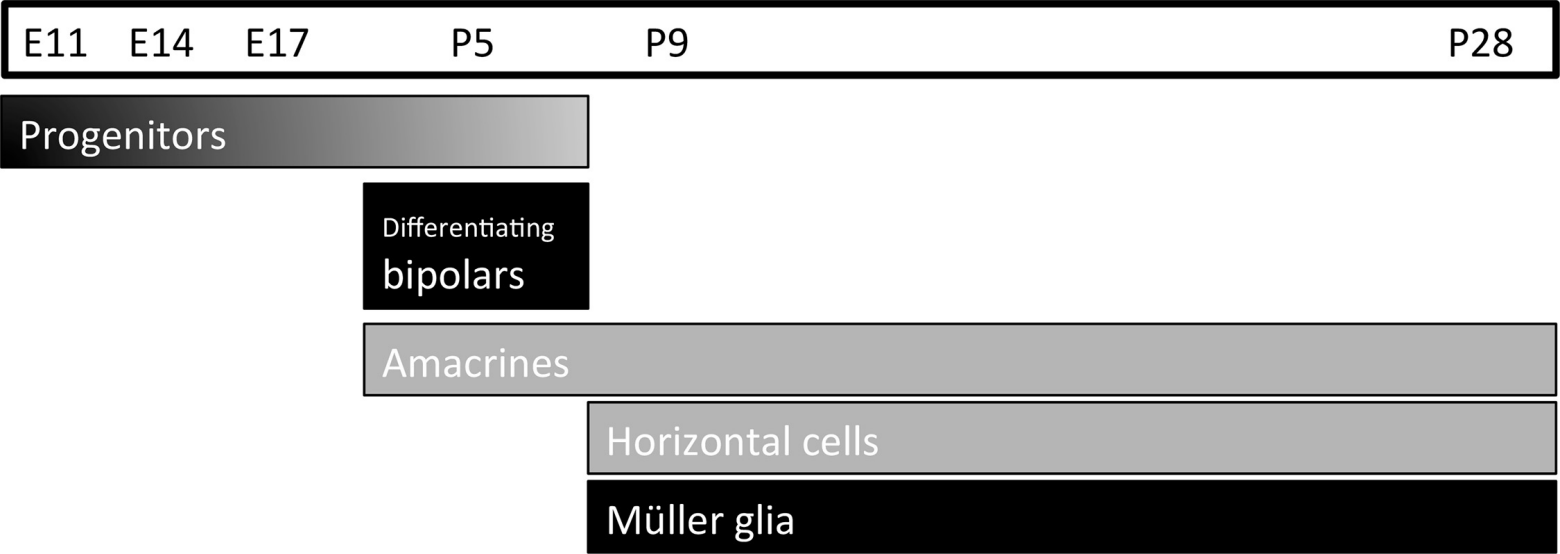
Expression of QKI during retinal development. During embryonic stages, QKI is expressed in progenitors. In the P5 retina, QKI is expressed in progenitors, differentiating bipolar cells, and amacrine cells. After P9 and until maturation, QKI is expressed predominantly in Müller glia and weakly in horizontal and amacrine cells.

In summary, our data show that QKI, an RNA-binding protein, was expressed predominately in Müller glia, and weakly in the early differentiating neurons, horizontal and amacrine cells in the mouse retina throughout late postnatal development (Fig 7). We did not detect any QKI signals in the photoreceptor layer at any developmental stages. QKI was expressed in the differentiating bipolar cells and retinal progenitors during early retinal development (S1 Fig). In the CNS other than the retina, QKI was detected in proliferating cells and glial cells, but not in neuronal cells. Müller glial cells have been recognized as being similar to retinal progenitor cells [40,41]. In retinal development, we showed that QKI was substantially expressed in Müller glia and progenitors, transiently in differentiating bipolar cells, and weakly in horizontal and amacrine cells. Therefore, QKI may contribute to maintaining retinal progenitors, disappearing when neuronal cells start to differentiate. We did not detect any QKI expression in photoreceptors but did find weak and transient expression in horizontal, amacrine, and developing bipolar cells. Thus, QKI may contribute to differential neuronal cell fate determination. Our data could not exclude the possibility of QKI expression in a subset of ganglion cells but we suspect that the localization of QKI-positive cells in the GCL results from displaced amacrine cells.

Among QKI isoforms, we found that QKI-5 predominates in the adult mouse retina (Figs 1 and 2E). Examination of the retinas of *qk*^*v*^ mutant mice did not detect significant reductions of QKI proteins in the retina, however, there were no significant reductions of QKI isoforms including QKI-5 in the q/q mutant retina compared with the q/w control. We also did not see any changes in morphology and cell specific markers in q/q mutant retinas by immunohistochemistry (data not shown). In contrast, QKI-6 and -7, but not QKI-5, were reduced in the q/q mutant optic nerve demonstrating an effect of the *qk*^*v*^ mutation. It is reported that QKI is required for oligodendrocyte cell fate and myelination [20,42,43], and our results also suggest that reductions of QKI-7, in addition to the previously known QKI-6 reduction, could be a cause of the optic nerve defect in q/q mutant mice [1,10]. Since the q/q mutant retina has no reduction in QKI isoforms and QKI-deficient mice are embryonic lethal at an early stage (between E9.5 and 10.5) [12], conditional knockout experiments will be necessary to reveal how QKI contributes to the maintenance of progenitors and the specification of both neuronal and glial cells during retinal development.

## Supporting Information

S1 Fig Specificities of anti-QKI antibody in the developmental retina. Immunodeletion assays were performed at P5 (A) and P9 (B) retina. Far left is control without primary antibody. Immunostaining with anti-QKI antibody showed strong signals (middle left). The anti-QKI antibody immunodepleted by incubation with GST–QKI (far right) significantly decreased the signals, whereas the anti-QKI antibody immunodepleted with GST showed strong signals by immunostaining (middle right). Scale bar is 50 μm.
(TIF)

S1 Table Cell types immunopositive for various antigens during the respective developmental stages. Parentheses show reference numbers.
(TIF)
