# Supplementary figures and images for: Expression of Quaking RNA-Binding Protein in the Adult and Developing Mouse Retina
Source: PLoS One. 2016 May 19;11(5):e0156033. doi: 10.1371/journal.pone.0156033 (PMC4873024; doi:10.1371/journal.pone.0156033)

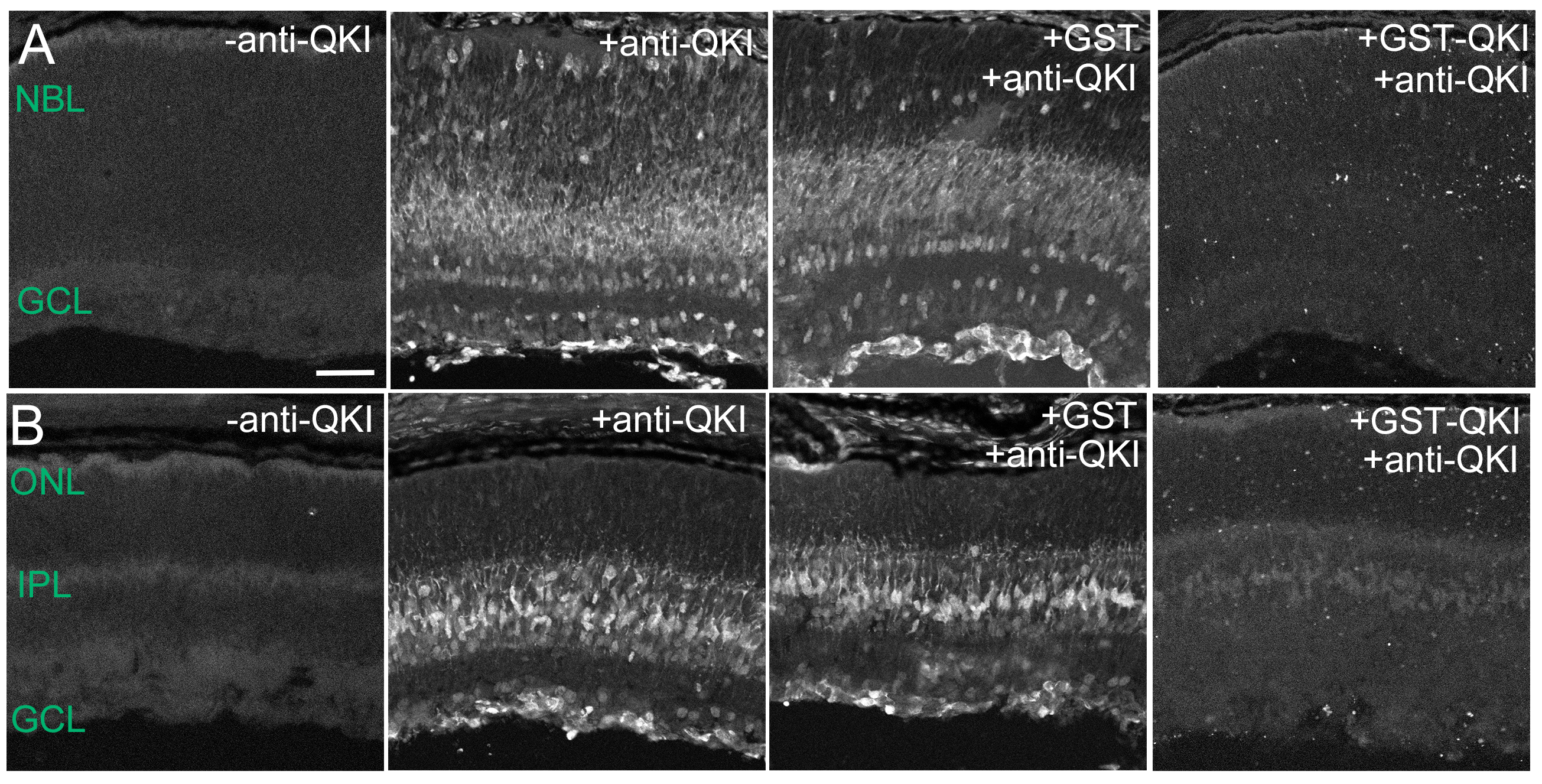

Supplement: S1 Fig — Immunodeletion assays were performed at P5 (A) and P9 (B) retina. Far left is control without primary antibody. Immunostaining with anti-QKI antibody showed strong signals (middle left). The anti-QKI antibody immunodepleted by incubation with GST–QKI (far right) significantly decreased the signals, whereas the anti-QKI antibody immunodepleted with GST showed strong signals by immunostaining (middle right). Scale bar is 50 μm. (TIF) [file pone.0156033.s001.tif]

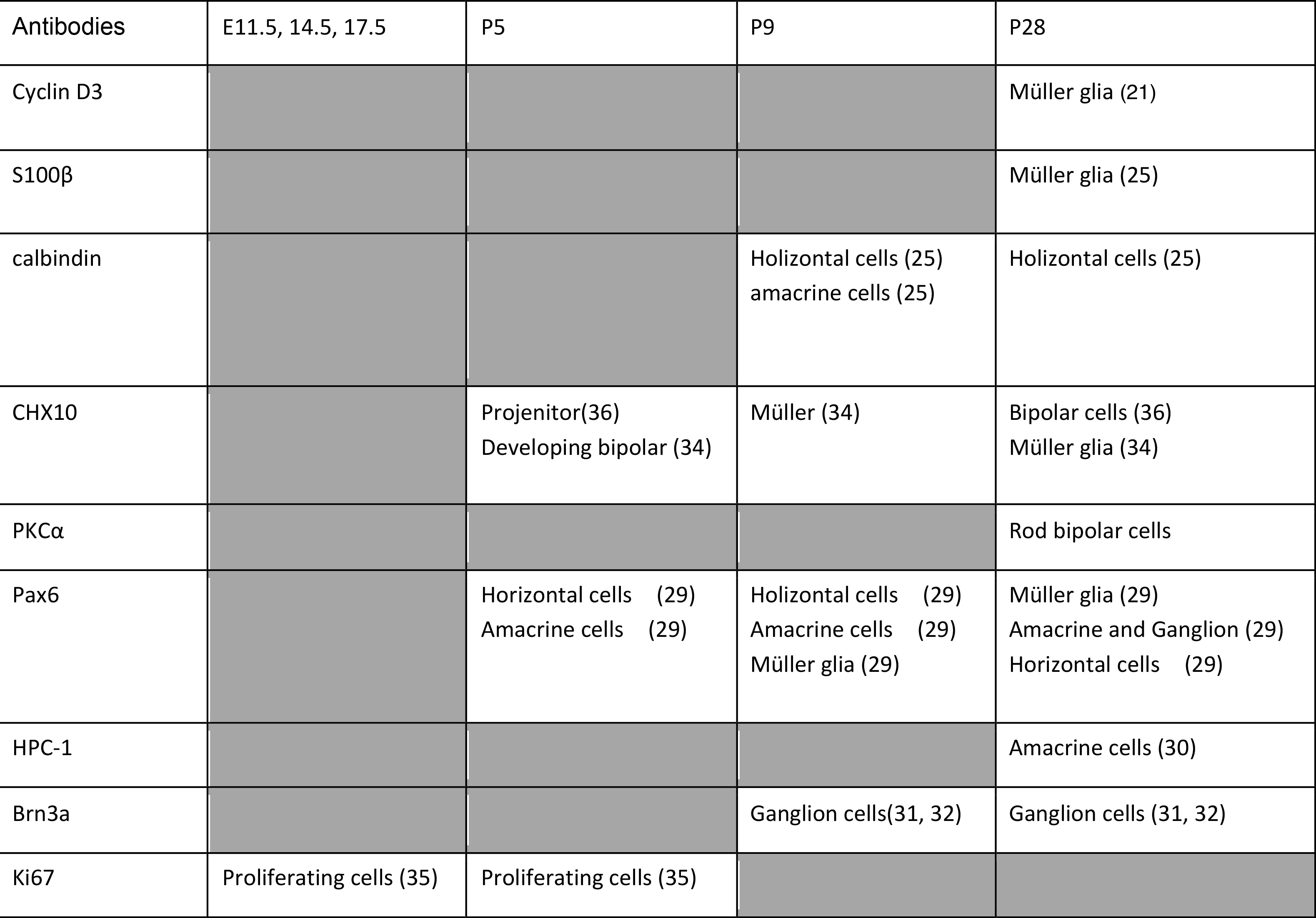

Supplement: S1 Table — Parentheses show reference numbers. (TIF) [file pone.0156033.s002.tif]
